# Decreased Immune Response in Alexithymic Women: A One-Year Longitudinal Study

**DOI:** 10.3389/fpsyt.2021.756031

**Published:** 2021-12-20

**Authors:** Olivier Guilbaud, Claire Perrin, Florence Curt, Gérard Chaouat, Corinne Dugré-Le Bigre, Martine Strebler, Catherine Touitou, Maurice Corcos

**Affiliations:** ^1^Département de Psychiatrie de l'Adolescent et du Jeune Adulte, Institut Mutualiste Montsouris, Paris, France; ^2^Consultation Thérapeutique Enfants et Adolescent, Renens, Switzerland; ^3^Inserm U 782, Hôpital Antoine Béclère, Clamart, France; ^4^Département de Biologie de l'Institut Mutualiste Montsouris, Paris, France

**Keywords:** alexithymia, cytokines, immunity, psychosomatic medicine, psychoneuroimmunology

## Abstract

Although previous cross-sectional studies suggested significantly dysregulated immune response in alexithymia, there is a lack of longitudinal studies. We sought to determine the reliability of the reported relationship between alexithymia and decreased immune response in a longitudinal study. Thirty-eight healthy women who had participated in a cross-sectional study were recontacted 1-year later. Of this sample, 26 were finally included: 13 females who had been found to be alexithymic, and 13 females who were classified as non-alexithymic under the 20-item Toronto Alexithymia Scale during the first phase of the study. A year later, they were still healthy women without any psychiatric disorders, their ages now ranging from 19 to 28 years old. Lymphocyte subset counts (CD4, CD8), *in vitro* production of interleukin 1β (IL-1β), IL-2, IL-4, and IL-10 by phytohemagglutinin stimulated peripheral blood lymphocytes, as well as serum cortisol levels, were compared between women with and without alexithymia. One-year later, alexithymic women still had significantly lowered *in vitro* production of IL-2 and IL-4, with lowered IL-2/IL-10 ratio and a reduced percentage of CD4. This is the first ever published study assessing cytokine production during a follow-up **of alexithymics**. Although our results should be interpreted with caution due the small sample size, they suggest a sustained reduction in both major type 1 and type 2 cytokines while the former seems to be more affected. The potential long-term health impact, if any, is still to be determined.

## Introduction

Alexithymia is a clinical description coined by Sifneos (1) in 1972 from the Greek for lack (a), word (lexis) and emotion (thymos) to mean “no words for feelings.” It is characterized by a set of cognitive-affective deficits, notably inaccuracy in identifying and describing emotions; difficulty in distinguishing between feelings and bodily sensations of emotional arousal; paucity of fantasies; and an externally oriented cognitive style. Although it was initially associated with so-called psychosomatic disease (2–4), alexithymia appears not to be specific to these diseases. It has been observed in depressed mood, anxiety disorders as well as psychological trauma, dissociation, with a particular attachment style (5, 6). Nevertheless, a stress-alexithymia hypothesis has been proposed (7–11), with empirical evidence supporting the premise that alexithymic characteristics influence the physiological stress response (9–11) and immunity (7, 8). Alexithymia has been associated with higher tonic or baseline levels of sympathetic activity and lower sympathetic reactivity during acute stress (9–11) as well as perturbation of the hypothalamo–pituitary–adrenocortical (HPA) axes (12–14). These subjects may display a dissociation between subjective and physiological stress responses. The decoupling response between feeling state and physiological arousal may increase alexithymic individuals'risk for stress related illness (7–11). In this specific set of cognitive-affective deficits there might be reduced or unnoticed reports of stress coupled with impaired autonomic, neuroendocrine, and behavioral responsivity. Indeed, alexithymics may be proned to chronically impaired sympathetic and neuroendocrine (notably HPA axis) activation during or prior to stress with impaired immune response. Although there is a lack of study evaluating the immune response during standardized stress test in alexithymics, several studies at baseline exhibited impaired immunity in these subjects (15–28).

In a cross-sectional study (17) comparing alexithymics vs. non-alexythymics in a sample of healthy young women, we observed that the alexithymic subjects exhibited significantly decreased production of interleukin1β (IL-1β), IL-2, and IL-4 by phytohaemagglutinin stimulated peripheral blood lymphocytes, associated with reduced ratios of Th1/Th2 (IL-2/IL-10) and of CD4/CD8, as well as reduced CD4 percentages, regardless of anxiety or depression levels. In this present study, we seek to determine the reliability of the reported relationships between alexithymia and decreased immune response. To this end, our previous study was extended to include a follow-up assessment 1-year later.

## Materials and Methods

### Participants and Procedure

The study sample was drawn from the same female university students as in the cross-sectional study (17), and, as in the cross-sectional study, all students participated at least 6 weeks before or after their academic examinations. As before, the exclusion criteria were the presence of current or lifetime experience of any DSM-IV axis I disorder as assessed by the Mini International Neuropsychiatric Interview (M.I.N.I.) (29); any autoimmune or inflammatory disease; recent influenza or recent infectious disease (within the past 4 weeks); and the use of any psychotropic, anti-inflammatory or immunosuppressive medications during the past 4 weeks as evaluated by medical report and examination. Subjects had to stop drinking and also abstain from caffeine for at least 3 days before coming to our laboratory. As 1-year earlier, the blood sample was collected for each woman during the luteal phase of their menstrual cycle. The dates of their periods and the length of their cycles were requested to summon them during the luteal period of their menstrual cycle.

For this 1-year longitudinal study, we recalled only those who had measurements of cytokine production, i.e., the 38 healthy young women (18 alexithymic and 20 non-alexithymic) who were recruited during the research carried out in 2004–2005 (17). A year later, participants were still healthy female university students, with ages now ranging from 19 to 28 years old. Of this sample, only 28 women attended 1-year later. Seven could not be recalled by phone or mail, two refused to come, and one failed to attend for her appointment. Of the 28 subjects, two met the exclusion criteria. One was under corticosteroid therapy and the other was suffering from inflammatory disease (with a CRP of 34). A total of 26 subjects were included 1-year later from the initial sample of 38. Among this sample, 13 had been initially diagnosed as alexithymic and 13 as non-alexithymic. The assessment of alexithymia was performed only at baseline. Subjects were included again 1-year later (CP). The investigator (CP), in a double-blind study (i.e., the investigator as well as the participants were blind to the TAS-20 scores during the entire study), gave all participants a written and oral explanation of the study. The research was approved by the Paris-Hospital Hotel Dieu Ethics Committee. All participants agreed to participate in this study, gave their written informed consent, and received 20 euros compensation.

### Assessment Tools

The absence of a current/past major depressive episode, anxiety disorders or any other psychiatric disorders according to DSM-IV was assessed again, using the M.I.N.I. (29, 30). It is a tool for assessing major diagnostic disorders according to DSM-IV criteria. It is a semi-structured interview. It was administered to the participants by the same evaluator (CP) who was trained in its use. The Hospital Anxiety and Depression Scale (HADS), a well-validated 14-item self-screening questionnaire (31), was used to assess rates of anxiety symptoms (HADS subscale for anxiety: HADS-A) and depressive symptoms (HADS subscale for depression: HADS-D). Because tobacco smoking is known to inhibit expression of certain cytokines (32), the Fagerström Test for Nicotine Dependence (FTND) (33) was used to evaluate use of nicotine. Note that in the cross-sectional study, alexithymia was rated using the French translation of the 20-item Toronto Alexithymia Scale (34, 35) and only non-alexithymics (TAS-20 ≤ 44) and alexithymics (TAS-20≥56) were initially recruited according to the TAS-20 (34) cut-off scores established for French-speaking samples (35) while sub-alexithymics were excluded (17). They were called back a year later to participate in this study. In the cross-sectional study, alexithymia was indeed considered as a categorical variable. The TAS-20 is a three-factor scale, namely, difficulty identifying feelings (DIF), difficulty describing feelings (DDF), and externally oriented thinking (EOT) (34, 35).

### Biological and Immunological Measurements

After an overnight fast, blood samples were collected in heparinized tubes between 8:00 a.m. and 9:00 a.m. and immediately transported to the laboratory. To determinate the WBC subset counts, the total number of WBC and leukocyte differential counts were calculated with a Coulter counter (CELL-DYN 4000; ABBOTT). Enumeration by flow cytometry with FACS count (Becton Dickinson) included the following cells: two types of T cell subsets CD4, CD8.

Serum levels of cortisol were measured using a competitive enzyme immunoassay which was performed entirely within the ST AIA-PACK CORT (Tosoh Bioscience).

### Assessment of Cytokine Production by PHA-Stimulated Lymphocytes

Lymphocytes were separated on a 25 ml Ficoll layer, cushioned by a 5 ml RPMI 1640 culture medium. This was done by spinning for 20 min at 1,200 rpm (g) in a Hereaeus centrifuge with swing out buckets at 4°C. Lymphocytes which were collected at the interface were washed twice in culture medium before being counted for viability using the Trypan blue dye exclusion test. Viability was usually well above 90%. 5 × 10 cells /ml were then cultured in 25 ml ventilated culture flasks (Costar, or equivalent) in a Fetal Calf Serum (FCS) supplemented culture medium, at 37°C in a 5% CO_2_ humid incubator, in the presence of phytohemagglutinin A (PHA P, Wellcome) at a final concentration of 2.5 μg/ml; for 48 h. All cultures were performed in RPMI 1640 with Glutamax (Gibco Life sciences) supplemented with 1% sodium bicarbonate, 1% penicillin streptomycin (both from Gibco), and 25 μl/500 ml mercaptoethanol (Sigma). This culture medium was itself supplemented with 10% heat-inactivated FCS. At the end of this period, culture supernatants were harvested by lymphocyte centrifugation and kept at −20° until ELISA assay. Commercial kits (R&D Systems, France) were used for all ELISA assays. Samples were tested in duplicate in the same assay. Inter- and intra-assay variabilities were always <10%. The lower limit of detectability was 1 pg/ml for IL-1β, 7 pg/ml for IL-2, 0.03–0.22 pg/ml for IL-4, 3.9 pg/ml for IL-10.

### Data Analyses

Data were analyzed using SPSS (27.00) software. The data were examined for normal distributions with the Shapiro-Wilk test. For normal distributions, *t*-tests were used. In the absence of normal distributions non-parametric tests (Mann and Whitney Wilcoxon) were performed. Most parameters were normally distributed with the exception of cytokine production. Differences in cell counts and serum concentrations of cortisol were determined using *t*-tests; for cytokine production, Mann and Whitney Wilcoxon tests were used according to the distribution. The results are presented as mean ± standard deviation. The effect size r was calculated for Wilcoxon tests (case of independent two-samples test), and Cohen's *d* for *t*-tests. The *r*-value varies from 0 to close to 1. The interpretation values for *r* commonly in published litterature and on the internet are: 0.10– < 0.3 (small effect), 0.30– < 0.5 (moderate effect), and ≥0.5 (large effect).

## Results

### Sociodemographic and Psychopatholical Data

There were no differences in the sociodemographic and psychopathological characteristics (age, HADS subscales for anxiety and depression, or the rate of alexithymia) for the subjects lost to follow-up compared to the 26 subjects who were included both in the cross-sectional study and 1-year later in the longitudinal study.

One-year later, there was still no difference between alexithymics (*n* = 13) and non-alexithymics (*n* = 13) regarding age (21.08 vs. 21.92; *p* = 0.42) and none of the subjects were suffering from tobacco addiction according to the Fagerström test. What is worthy of note is that there were no longer any differences (see Table 1) between the two groups in terms of the HADS subscale for anxiety (HADS-A, *p* = 0.28) or the HADS subscale for depression (HADS-D; *p* = 0.42).

**Table 1:** Scores of HADS for anxiety and depression of alexithymic women vs. non-alexithymic women (1-year later).

**Characteristics**	**Alexithymic women**	**Non-alexithymic women**	**Analysis**		
	**Mean (S.D)**	**Mean (S.D)**	***t***	***ddl***	***p***
	***n* = 13**	***n* = 13**			
HAD depression	1.85 (1.99)	1.31 (1.37)	*t* = −0.93	24	*p* = 0.42
HAD anxiety	5.31 (2.29)	4.08 (3.38)	*t* = −1.25	24	*p* = 0.28

*S.D., standard deviation; HADS, hospital anxiety depression scale*.

### Immunological Measures

As shown in Table 2, compared with the non-alexithymics, the alexithymic subjects still had significantly lowered percentages of CD4+ T cells (*p* = 0.039; Cohen's *d* was of 0.882, 95% CI [0.066–1.681]) 1-year later. On the other hand, although there was no longer any significant difference, there was a tendency toward decreased CD4/CD8 levels in alexithymic subjects (*p* = 0.1). We did not observe, 1-year later, any significant differences in terms of the percentage of lymphocytes or the neutrophil-to-lymphocyte ratio (see Table 2).

**Table 2:** Immunological characteristics and serum levels of cortisol of alexithymic women vs. non-alexithymic women who were recalled and included (1-year later).

**Characteristics**	**Alexithymic women**	**Non-alexithymic women**	**Analysis**		
	**Mean (S.D)**	**Mean (S.D)**	***t***	***ddl***	***p***
	***n* = 13**	***n* = 13**			
Cortisol	219.31 (72.44)	248.31 (58.69)	*t* = 1.12	24	*p* = 0.27
Polynuclear (cells/mm^3^)	3546.20 (213.45)	3123.07 (115.91)	*t* = −0.63	24	*p* = 0.70
Polynuclear %	53.76 (9.67)	55.06 (7.14)	*t* = 0.39	24	*p* = 0.70
Lymphocytes (cells/mm^3^)	2138.50 (442.6)	1969.20 (540.65)	*t* = −0.83	24	*p* = 0.38
Lymphocytes %	36.40 (8.96)	35.28 (7.37)	*t* = −0.35	24	*p* = 0.78
Tcells (CD3+) (cells/mm^3^)	1645.77 (374.33)	1600.15 (520.75)	*t* = −0.25	24	*p* = 0.80
T cells (CD3+) %	76.85 (7.65)	81.31 (5.79)	*t* = 1.67	24	*p* = 0.11
CD4+ T cells (cells/mm^3^)	991.85 (246.69)	1029.61 (383.24)	*t* = 0.29	24	*p* = 0.77
CD4+ T cells %	46.08 (5.96)	51.69 (6.75)	*t* = 2.25	24	*p* = 0.039
CD8+ T cells (cells/mm^3^)	590.69 (145.12)	511.07 (169.29)	*t* = 1.28	24	*p* = 0.21
CD8+ T cells %	27.54 (4.42)	26.46 (5.56)	*t* = −0.55	24	*p* = 0.59
CD4/CD8 ratio	1.72 (0.36)	2.06 (0.62)	*t* = 1.73	24	*p* = 0.10
Neutrophiles/Lymphocytes	1.67 (0.90)	1.63 (0.56)	*t* = −0.12	24	*p* = 0.90

One-year later, the alexithymic group still exhibited significantly reduced production of IL-2 and IL-4 PHA-stimulated lymphocytes (all *p*-values ≤ 0.034; see Table 3) with moderate effect size (respectively *r* = 0.425 and *r* = 0.420). In addition, in comparison with the non-alexithymics, alexithymics still had lowered IL-2/IL-10 ratio (*p* = 0.037) but there was no longer any significant difference concerning *in vitro* production of IL-1β (see Table 3).

**Table 3:** Cytokines produced by PHA stimulated lymphocytes (1-year later).

**Characteristics**	**Alexithymic women**	**Non-alexithymic women**	**Analysis**		
	**Mean (S.D)**	**Mean (S.D)**	***W***	***Z***	***p***
	***n* = 13**	***n* = 13**			
IL-1 β	142.89 (170.58)	83.87 (91.49)	*W* = 152.00	−1.20	*p* = 0.23
IL-2	46.62 (68.89)	325.23 (459.96)	*W* = 130.00	−2.12	*p* = 0.034
IL-4	0.08 (0.19)	0.43 (0.59)	*W* = 140.00	−2.14	*p* = 0.032
IL-10	43.55 (31.71)	57.41 (73.25)	*W* = 175.50	0.00	*p* = 1.0
IL-2/IL-10	1.30 (1.35)	7.11 (7.85)	*W* = 110.00	−2.09	*p* = 0.037

*For technical reasons, one aliquot was missing for IL-2 (n = 25). S.D, standard deviation; PHA, phytohemaglutinin A; IL, interleukin*.

### Associations Between Immune Parameters and HADS Subscale Scores

Decreases in IL-2, IL-4 productions, and CD4 percentage were strictly independent of anxiety and depression rates, as there were no more significant differences between the two groups given the HADS scores for anxiety and depression (see Table 1).

### Cortisol Measures

There were still no significant between-group differences in serum cortisol levels (see Table 2).

## Discussion

In line with our previous cross-sectional study (17), healthy women who were diagnosed as alexithymic according to the TAS-20 scale still had lowered *in vitro* production of IL-2 and IL-4 with lowered IL-2/IL-10 ratio and reduced percentage of CD4 1-year later. Besides, there were no longer any differences between alexithymic and non-alexithymic groups regarding rates of anxiety and depression. These results also confirm the analyses performed a year earlier (17), showing a significant reduction in IL-2 and IL-4 production in alexithymic women regardless of anxiety and depression. These results seem to suggest a specific immunosuppressive effect of alexithymia on Th1 and Th2 cytokine productions regardless of anxiety and depression. Type 1 T helper (Th1) cells produce interferon-gamma, interleukin (IL-2), and tumor necrosis factor (TNF-β) which induce macrophage activation and induce cell-mediated immunity, whereas type 2 cells produce IL-4, IL-5, IL-10, IL-13, and promote humoral immunity (36). It could be argued that the lowered IL-2/Il-10 production and the decreased percentage of CD4 still suggest a more pronounced reduction of Th1 immunity. In previous reports (7, 8), we hypothesized that alexithymia was associated with a predominance of depressed cell-mediated immunity and a Th1/Th2 ratio skewed toward Th2 response. Nevertheless, we must also recognize that Th2 response is still impaired 1-year later, even if it is to a lesser degree.

One-year later, we did not find any significant IL-1β *in vitro* production difference between groups. The fact that IL-1β was no more diminished 1-year later could be due to the smaller sample size (26 subjects 1-year later vs. 38 subjects during the cross-sectional study when considering cytokine production) and perhaps to the fact that this cytokine is less specific to the immune dysregulation observed in alexithymia.

To our knowledge, this is the first longitudinal study ever published assessing the immune parameters (notably cytokines) in alexithymics compared to non-alexythymics.

Temoshok et al. (18) found, in a basic sample of 200 HIV-infected subjects, a significant association between alexithymia and lower stimulated production of HIV-MIP-1α. MIP-1α is the chemokine macrophage inflammatory protein which is known to play a key role in the protection from infection (37) and to stimulate macrophages to secrete pro-inflammatory cytokines (38). Also, McIntosh et al. (15) observed, in a sample of HIV survivors, a higher HIV viral load in alexithymic subjects (*n* = 93) comparatively to non-alexithymic subjects (*n* = 74). These results combined with ours suggest a decreased immune response in subjects afflicted with alexithymia.

A brief review of the literature shows that most studies evaluating immunity in alexithymics are based on immunocyte counts or on the measurement of circulating levels of cytokines. Several other authors found decreased immunity in alexithymics in cross-sectional studies.

Todarello et al. (21, 22) and Dewaraja et al. (20) found that individuals with alexithymia had lower counts of numerous lymphocytic subsets. In a categorical approach, Dewaraja et al. (20) observed significantly reduced cytotoxic natural killer subsets (CD57–CD16+ cells) in healthy men with alexithymia compared to men without alexithymia. In a sample of patients who were unaware that they are suffering from cervical dysplasia, Todarello et al. (21, 22) found significantly diminished levels of almost all lymphocyte subsets (CD2, CD3, CD4) in patients with alexithymia.

When considering serum cytokine levels, results are still contradictory. For instance, Mandarelli et al. (16) observed significantly lowered serum levels of IL-4 and Il-6 in alexithymic (*n* = 22) compared to non-alexithymic (*n* = 37) patients undergoing upper endoscopy, regardless of stress, anxiety, and depression. In 24 male and female patients suffering from Somatoform Disorder (SFD), Pedrosa Gil et al. (19), found a significant negative correlation between alexithymia and serum levels of IL-2 receptor while they observed significant positive correlations between alexithymia and serum levels of IL-6, IL-10, and Immunoglobulin E. These authors suggested that patients suffering from SFD with clinically significant alexithymia exhibit a reduction in Th1-mediated immune function and an increase in the activation of the Th2 immune function. In a preliminary study (28), we observed a significant positive correlation between serum levels of IL-4 and alexithymia; however we found the opposite result when we studied *in vitro* production of IL-4 by phytohaemagglutinin stimulated peripheral blood lymphocytes.

As we stated before (17), analysis of the results in terms of circulating levels of cytokines remains complex. Cytokines derive primarily from a range of synthetic sources: the immune system, the adipocytes (39), the intestinal mucous membrane and at much lower levels (due to the blood brain barrier) from the central nervous system (40). Moreover, the circulating levels of cytokines are not always congruent with their *in vitro* production (41) (see also the Limitations section).

Other studies have observed increased serum levels of pro-inflammatory cytokines in alexithymia. Bossu et al. (25) observed a strong significant correlation between IL-18, a member of the IL-1 family, and alexithymia amongst patients with right hemisphere lesions after acute stroke. Vadaca et al. (26) found a significant positive correlation between TNFα, IL-6, and alexithymia in patients with lupus erythematosus and rheumatoid arthritis (RA); Bruni et al. (27) observed a significant correlation between serum levels of TNF and alexithymia in RA; and Uher et al. (23) observed a significant positive correlation between cerebrospinal fluid levels of IL-8 and alexithymia and anxiety in non-inflammatory neurological disorder.

From all these studies, two broad models can thus be identified: an overall decrease in immunity with a more or less well-preserved Th2 immunity, and an acute-phase proteins reaction (42, 43) with increased levels of pro-inflammatory cytokines (IL-1, IL-6, TNF). The first model seems to reproduce the effects of subchronic stress on immunity: suppressing or dysregulating innate and adaptive immune responses by altering the Type 1-Type 2 cytokine balance, inducing low-grade chronic inflammation, and suppressing both the numbers and the function of immunoprotective cells (44). Segerstrom and Miller (45) in a meta-analysis found that chronic stressors such as dementia care-giving, living with a handicap or unemployment were associated with suppression of both cellular and humoral measures. Natural and specific immunity were negatively affected, as were Th1 (e.g., T cell proliferative responses) and Th2 (e.g., antibody to influenza vaccine) parameters except for antibody to latent virus (45). The second model seems to be similar to the assumed immune activation observed in patients with major depression characterized by an increase in pro-inflammatory cytokines that seems to correlate with severity of illness and measures of hypothalamic–pituitary–adrenal (HPA) axis hyperactivity (46, 47). It is also characterized by a switch of the pro-inflammatory and anti-inflammatory cytokine balance toward a pro-inflammatory imbalance with accompanying altered cell-mediated immunity (48). From their results, Honkalampi et al. (24) suggest that there may be some difference in the mechanism leading to proinflammatory states when comparing depression and alexithymia, i.e., alexithymic individuals may have a weakened anti-inflammatory buffer capacity, whereas those with depression appear to show a pronounced proinflammatory state. It is also known that in some chronic diseases, such as chronic low back pain and fibromyalgia syndrome, alexithymic patients have higher scores of pain intensity and perception than non-alexithymic ones (49). This could be related to the cytokines' burden. Marchi et al. (49) demonstrated that alexithymia plays a more important role in influencing pain, regardless of the presence and severity of comorbid anxiety or depression.

Because, we failed twice (17) to find any significant increase in pro-inflammatory cytokines such as IL-1β, our results in young healthy women are in line with the first model. However, our results reflect the state of the immune system and do not necessarily reflect the neuroimmunomodulation process and what is happening in the nervous system. Alexithymic status seems to decrease immune response, particularly in terms of Th1 immunity (and to a lesser extent Th2) when considering the production of certain cytokines (IL-2, IL-4, and IL-10) for a long period.

Are alexithymics, because of their inability to express and recognize their emotions, more prone to subchronic stress? Subchronic unnoticed emotional distress in healthy women with alexithymia may be associated with impaired immunity and higher physiological stress responses as stated in the stress-alexithymia hypothesis (9, 10). Are the two models mentioned above two sides of the same coin? Is the activation of pro-inflammatory cytokines a compensatory mechanism of overall weakened immunity, or is it a totally different mechanism observed in patients prone to develop autoimmune disease? It should also be remembered that chronic stress has been also associated with increased pro-inflammatory cytokines while decreasing the anti-inflammatory ones (50). For instance, Miller et al. (51) reported a diminish capacity of a synthetic glucocorticoid hormone to suppress *in vitro* production of the pro-inflammatory cytokine interleukin-6 in subjects afflicted with chronic stress.

## Limitations

Our study population consisted of young healthy women, which might explain the difference from some other studies, since it has been reported that age (52), gender (53), and disease (45) (such as inflammatory response) change immune response.

We only assessed *in vitro* cytokine production, which provides useful information regarding the functioning of the immune system (39) but is closely related to the activation of the peripheral immune system. Moreover, several studies (40) have reported discrepancies between cytokine levels and cytokines produced by lymphocyte activation. For instance, Steptoe et al. (41) report robust effects for increased levels of circulating IL-6 and IL-1β following acute stress while the stimulated cytokine production was less consistent.

The small size of the sample requires that our results be interpreted with caution, and also requires that this longitudinal study be replicated on a larger scale.

## Conclusion

To our knowledge, this is the first ever published study assessing cytokine production 1-year later in alexithymics. In line with previous research, our finding that the relationship between alexithymia and decreased production of cytokines is maintained over a 12-month period corroborates the notion that immune function is impaired for a longer period than for non-alexithymic subjects. One-year later, alexithymics still exhibited significant decreased production of IL-2, IL-4, and a reduced percentage of CD4. To be alexithymic seems to be a risk factor for a prolonged impaired immunity (especially but not exclusively Th1) at least 1-year later. Obviously, we need more longitudinal studies in this area of research, with larger samples, to better understand the possible immune dysfunction in alexithymic subjects and its potential long-term health impact notably the risk of infectious diseases and immunological disorders as seen under chronic stress conditions (44, 45, 50, 51, 54).

## Data Availability Statement

The original contributions presented in the study are included in the article/supplementary files, further inquiries can be directed to the corresponding author/s.

## Ethics Statement

The studies involving human participants were reviewed and approved by Paris-Hospital Hotel Dieu Ethics Committee. The patients/participants provided their written informed consent to participate in this study.

## Author Contributions

All authors listed have made a substantial, direct, and intellectual contribution to the work and approved it for publication.

## Dedication

This article is dedicated to the memories of Claire Perrin and Gerard Chaouat.

## Conflict of Interest

The authors declare that the research was conducted in the absence of any commercial or financial relationships that could be construed as a potential conflict of interest.

## Publisher's Note

All claims expressed in this article are solely those of the authors and do not necessarily represent those of their affiliated organizations, or those of the publisher, the editors and the reviewers. Any product that may be evaluated in this article, or claim that may be made by its manufacturer, is not guaranteed or endorsed by the publisher.

## References

[1] Sifneos PE. The prevalence of ‘alexithymic' characteristics in psychosomatic patients. Psychother Psychosom. (1973) 22:255–62. 10.1159/000286529 4770536

[2] Nemiah JC Sifneos PE. Affect and fantasy in patients with psychosomatic disorders. In: Hill OW, editor. Modern Trends in Psychosomatic Medicine. London: Butterworths. (1970). p. 26–34.

[3] Todarello O Taylor GJ Parker JD Fanelli M. Alexithymia in essential hypertensive and psychiatric outpatients: a comparative study. Psychiatry Res. (1995) 39:987–94. 10.1016/0022-3999(95)00506-4 8926608

[4] Dirks JF Robinson SK Dirks DL. Alexithymia and the psychomaintenance of bronchial asthma. Psychother Psychosom. (1981) 36:63–71. 10.1159/000287527 7330162

[5] Guilbaud O Berthoz S Dupont M-E Corcos M. Alexithymia and psychosomatic disorders. EMC Psychiatr. (2009) 37-400-D-20:1–13. 10.1016/S0246-1072(09)48766-2

[6] Zdankiewicz-Scigała E Konrad Scigała D. Attachment style, early childhood trauma, alexithymia, and dissociation among persons addicted to alcohol: structural equation model of dependencies. Front Psychol. (2020) 10:2957. 10.3389/fpsyg.2019.02957 32038366 PMC6993624

[7] Guilbaud O., Corcos M., Jeammet P. Alexithymia, stress and immunity. In: Plotnikoff N, editor. Cytokines: Stress and Immunity. 2nd ed. Boca Raton, FL: Taylor & Francis (2006). p. 101–9.

[8] Guilbaud O. Corcos M, Hjalmarsson L, Loas G, Jeammet P. Is there a psychoneuroimmunological pathway between alexithymia and immunity? Immune and physiological correlates of alexithymia. Biomed Pharmacother. (2003) 57:292–5. 10.1016/S0753-3322(03)00085-4 14499176

[9] Martin JB. Pihl RO. The stress-alexithymia hypothesis: theoretical and empirical. Psychother Psychosom. (1985) 43:169–76. 10.1159/000287876 4034888

[10] Martin JB Pihl RO. Influence of alexithymic characteristics on physiological and subjective stress responses in normal individuals. Psychother Psychosom. (1986) 45:66–77. 10.1159/000287930 3786644

[11] Friedlander L Lumley MA Farchione T Doyal G. Testing the alexithymia hypothesis: physiological and subjective responses during relaxation and stress. J Nerv Ment Dis. (1997) 185:233–9. 10.1097/00005053-199704000-00003 9114808

[12] Cascino G Monteleone AM Marciello M Pellegrino F Ruzzi V Monteleone P. Alexithymia and cortisol awakening response in people with eating disorders. World J Biol Psychiatry. (2021) 22:546–51. 10.1080/15622975.2020.1844291 33135561

[13] Kajanoja J Karukivi M Mustonen P Scheinin NM Kortesluoma S Rodrigues AJ. Alexithymic traits and hair cortisol concentrations in pregnant women. Front Psychiatry. (2020) 11:421. 10.3389/fpsyt.2020.00421 32477193 PMC7237750

[14] Heinrichs M Wagner D Schoh W Soravia LM Hellhammer DH Ehlert U. Predicting postraumatic stress symptoms from pretraumatic risk factors: a 2 years prospective follow-up study in firefighters. Am J Psychiatry. (2005) 162:2276–86. 10.1176/appi.ajp.162.12.2276 16330591

[15] McIntosh RC Ironson G Antoni M Kumar M Fletcher MA Schneiderman N. Alexithymia is linked to neurocognitive, psychological, neuroendocrine, and immune dysfunction in persons living with HIV. Brain Behav Immun. (2014) 36:165–75. 10.1016/j.bbi.2013.10.024 24184475

[16] Mandarelli G Tarsitani L Ippoliti F Covotta F Zerella MP Mirigliani A. The relationship between alexithymia and circulating cytokine levels in subjects undergoing upper endoscopy. Neuroimmunomodulation. (2011) 18:37–44. 10.1159/000315529 20616574

[17] Guilbaud O Curt F Perrin C Chaouat G Berthoz S Dugré-Le Bigre C. Decreased immune response in alexithymic women: a cross-sectional study. Biomed Pharmacother. (2009) 63:297–304. 10.1016/j.biopha.2008.08.007 18824323

[18] Temoshok LR1 Waldstein SR Wald RL Garzino-Demo A Synowski SJ Sun L. Type C coping, alexithymia, and heart rate reactivity are associated independently and differentially with specific immune mechanisms linked to HIV progression. Brain Behav Immun. (2008) 22:781–92. 10.1016/j.bbi.2008.02.003 18346864

[19] Pedrosa Gil F Nickel M Ridout N Schwarz MJ Schoechlin C Schmidmaier R. Alexithymia and interleukin variations in somatoform disorder. Neuroimmunomodulation. (2007) 14:235–42. 10.1159/000112048 18073498

[20] Dewaraja R Tanigawa T Araki S Nakata A Kawamura N Ago Y. Decreased cytotoxic lymphocyte counts in alexithymia. Psychother Psychosom. (1997) 66:83–6. 10.1159/000289113 9097335

[21] Todarello O Casamassima A Daniele S Marinaccio M Fanciullo F Valentino L. Alexithymia, immunity and cervical intraepithelial neoplasia: replication. Psychother Psychosom. (1997) 66:208–13. 10.1159/000289136 9259044

[22] Todarello O Casamassima A Marinaccio M La Pesa MW Caradonna L Valentino L. Alexithymia, immunity and cervical intraepithelial neoplasia: a pilot study. Psychother Psychosom. (1994) 61:199–204. 10.1159/000288890 8066158

[23] Uher T Bob P. Cerebrospinal fluid IL-8 levels reflect symptoms of alexithymia in patients with non-inflammatory neurological disorders. Psychoneuroendocrinology. (2011) 36:1148–53. 10.1016/j.psyneuen.2011.02.006 21397404

[24] Honkalampi K Lehto SM Koivumaa-Honkanen H Hintikka J Niskanen L Valkonen-Korhonen M. Alexithymia and tissue inflammation. Psychother Psychosom. (2011) 80:359–64. 10.1159/000327583 21829048

[25] Bossù P Salani F Cacciari C Picchetto L Cao M Bizzoni F. Disease outcome, alexithymia and depression are differently associated with serum IL-18 levels in acute stroke. Curr Neurovasc Res. (2009) 6:163–70. 10.2174/156720209788970036 19534720

[26] Vadacca M Bruni R Cacciapaglia F Serino F Arcarese L Buzzulini F. Alexithymia and immunoendocrine parameters in patients affected by systemic lupus erythematosus and rheumatoid arthritis. Reumatismo. (2008) 60:50–6. 10.4081/reumatismo.2008.50 18432325

[27] Bruni R Serino FM Galluzzo S Coppolino G Cacciapaglia F Vadacca M. Alexithymia and neuroendocrineeimmune response in patients with autoimmune diseases. Preliminary results on relationship between alexithymic construct and TNF alpha levels. Ann N Y Acad Sci. (2006) 1069:208–11. 10.1196/annals.1351.018 16855147

[28] Corcos M Guilbaud O Paterniti S Curt F Hjalmarsson L Moussa M. Correlation between serum levels of interleukin-4 and alexithymia scores in healthy female subjects: preliminary findings. Psychoneuroendocrinology. (2004) 29:686–91. 10.1016/S0306-4530(03)00087-8 15041089

[29] Lecrubier Y Sheehan DV Weiller E Amorim P Bonora I Sheehan KH. The Mini International Neuropsychiatric Interview (MINI). A short diagnostic structured interview: reliability and validity according to the CIDI. Eur Psychiatry. (1999) 12:224–31. 10.1016/S0924-9338(97)83296-8

[30] Sheehan DV Lecrubier Y Sheehan KH Amorim P Janavs J Weiller E. The mini-international neuropsychiatric interview (M.I.N.I.): the development and validation of a structured diagnostic psychiatric interview for DSM-IV and ICD-10. J Clin Psychiatry. (1998) 59(Suppl 20):22–33; quiz 34–57. 9881538

[31] Zigmond AS Snaith RP. The hospital anxiety and depression scale. Acta Psychiatr Scand. (1983) 67:361–70. 10.1111/j.1600-0447.1983.tb09716.x 6880820

[32] Chen H Cowan MJ Hasday JD Vogel SN Medvedev AE. Tobacco smoking inhibits expression of proinflammatory cytokines and activation of IL-1R-associated kinase, p38, and NF-kappaB in alveolar macrophages stimulated with TLR2 and TLR4 agonists. J Immunol. (2007) 179:6097–106. 10.4049/jimmunol.179.9.6097 17947684

[33] Heatherton TF Kozlowski LT Frecker RC Fagerstrom KO. The Fagerstrom test for nicotine dependence: a revision of the Fagerstrom Tolerance Questionnaire. Br J Addict. (1991) 86:1119–27. 10.1111/j.1360-0443.1991.tb01879.x 1932883

[34] Bagby R Parker JDA Taylor GJ. The Twenty-item Toronto Alexithymia Scale I item selection and cross-validation of the factor structure ii convergent, discriminant, and concurrent validity. J Psychosom Res. (1994) 38:33–40. 10.1016/0022-3999(94)90006-X 8126688

[35] Loas G Otmani O Fremaux D Lecercle C Duflot M Delahousse J. External validity, reliability and basic score determination of the Toronto Alexithymia Scales (TAS and TAS-20) in a group of alcoholic patients. Encéphale. (1996) 22:35–40. 8681873

[36] Elenkov IJ Chrousos GP. Stress hormones, proinflammatory and antiinflammatory cytokines, and autoimmunity. Ann N Y Acad Sci. (2002) 966:290–303. 10.1111/j.1749-6632.2002.tb04229.x 12114286

[37] Garzino-Demo. Chemokines and defensins as HIV suppressive factors: an evolving story. Curr Pharm Des. (2007) 13:163–72. 10.2174/138161207779313696 17269925

[38] Fahey TJ Tracey KJ Tekamp-Olson P Cousens LS Jones WG Shires GT. Macrophage inflammatory protein 1 modulates macrophage function. J Immunol. (1992) 148:2764–9. 1573267

[39] Weisberg SP McCann D Desai M Rosenbaum M Leibel RL Ferrante AW Jr. Obesity is associated with macrophage accumulation in adipose tissue. J Clin Invest. (2003) 112:1796–808. 10.1172/JCI200319246 14679176 PMC296995

[40] Kronfol Z Remick DG. Cytokines and the brain: implications for clinical psychiatry. Am J Psychiatry. (2000) 157:683–94. 10.1176/appi.ajp.157.5.683 10784457

[41] Steptoe A Hamer M Chida Y. The effects of acute psychological stress on circulating inflammatory factors in humans: a review and meta-analysis. Brain Behav Immun. (2007) 21:901–12. 10.1016/j.bbi.2007.03.011 17475444

[42] Conti C Caraffa A Kritas SK Ronconi G Fulcheri M. Alexithymia and its relationships with inflammatory response mediated by IL-1 family members. J Biol Regul Homeost Agents. (2017) 31:21–8. 28337867

[43] De Berardis D Conti C Iasevoli F Valchera A Fornaro M Cavuto M. Alexithymia and its relationships with acute phase proteins and cytokine release: an updated review. J Biol Regul Homeost Agents. (2014) 28:795–9. 25620189

[44] Dhabhar FS. Effects of stress on immune function: the good, the bad, and the beautiful. Immunol Res. (2014) 58:193–210. 10.1007/s12026-014-8517-0 24798553

[45] Segerstrom SC Miller GE. Psychological stress and the human immune system: a meta-analytic study of 30 years of inquiry. Psychol Bull. (2004) 130:601–30. 10.1037/0033-2909.130.4.601 15250815 PMC1361287

[46] Maes M. The cytokine hypothesis of depression: inflammation, oxidative & nitrosative stress (IO&NS) and leaky gut as new targets for adjunctive treatments in depression. Neuro Endocrinol Lett. (2008) 29:287–91. 18580840

[47] Maes M. Evidence for an immune response in major depression: a review and hypothesis. Prog Neuropsychopharmacol Biol Psychiatry. (1995) 19:11–38. 10.1016/0278-5846(94)00101-M 7708925

[48] De Berardis D Conti C Iasevoli F Valchera A Fornaro M Cavuto M. Alexithymia and its relationships with acute phase proteins and cytokine release: an updated review. J Biol Regul Homeost Agents. (2014) 28:795–9. 25620189

[49] Marchi L Marzetti F Orrù G Lemmetti S Miccoli M Ciacchini R. Alexithymia and psychological distress in patients with fibromyalgia and rheumatic disease. Front Psychol. (2019) 10:1735. 10.3389/fpsyg.2019.01735 31417462 PMC6685004

[50] Tian R Hou G Li D Yuan TF A. A possible change process of inflammatory cytokines in the prolonged chronic stress and its ultimate implications for health. ScientificWorldJournal. (2014) 2014:780616. 10.1155/2014/780616 24995360 PMC4065693

[51] Miller GE Cohen S Ritchey AK. Chronic psychological stress and the regulation of pro-inflammatory cytokines: a glucocorticoid-resistance model. Health Psychol. (2002) 21:531–41. 10.1037/0278-6133.21.6.531 12433005

[52] Pettiford JN Jason J Nwanyanwu OC Archibald LK Kazembe PN Dobbie H. Age-related differences in cell-specific cytokine production by acutely ill Malawian patients. Clin Exp Immunol. (2002) 128:110–7. 10.1046/j.1365-2249.2002.01813.x 11982598 PMC1906374

[53] Aulock SV Deininger S Draing C Gueinzius K Dehus O Hermann C. Gender difference in cytokine secretion on immune stimulation with LPS and LTA. J Interferon Cytokine Res. (2006) 26:887–92. 10.1089/jir.2006.26.887 17238831

[54] Song H Fall K Fang F Erlendsdóttir F Lu D Mataix-Cols D. Stress related disorders and subsequent risk of life threatening infections: population based sibling controlled cohort study. BMJ. (2019) 367:l5784. 10.1136/bmj.l5784 31645334 PMC6812608

